# Influence of Five Different Commercially Available Mouthwashes on the Growth of Candida albicans Adhered to Customized Prefabricated Heat-Cured Denture Base Acrylic Resin Sheets: An In Vitro Study

**DOI:** 10.7759/cureus.58301

**Published:** 2024-04-15

**Authors:** Neethu Poyil, Pattathil Abdul Razak, Aysha Mohamed Ali KP, Deepthi Venugopalan, Amal Jassim, Athira Krishna K

**Affiliations:** 1 Department of Prosthodontics, Kannur Dental College, Kannur, IND; 2 Department of Prosthodontics, MES Dental College, Perinthalmanna, IND; 3 Department of Prosthodontics, Malabar Dental College and Research Center, Malappuram, IND; 4 Department of Prosthodontics, Sree Anjaneya Institute of Dental Sciences, Kozhikode, IND

**Keywords:** cetylpyridinium chloride, sabarauds dextrose agar (sda), heat-cured acrylic denture base, colony forming units (cfu), ph meter, - candida albicans

## Abstract

Background

The purpose of this in vitro investigation was to evaluate the impact of five distinct commercial mouthwashes on the development of *Candida albicans* that had been adhered to heat-cured acrylic resin sheets.

Methods

This in vitro investigation was carried out at the MES Medical College's Microbiology Department in Perinthalmanna, Kerala, India. A total of 72 heat-cured acrylic resin sheets, size 10 × 10 × 2 mm, were fabricated. After disinfection, all 72 acrylic sheets were placed in a flask containing a suspension of the standard strain of *Candida* species (American Type Culture Collection) and incubated at 37ºC for 24 hours. Then, the acrylic sheets were randomly divided into six groups, with each group containing 12 acrylic sheets. Group 1 was the control group to which no mouthwash was added. In group 2, Colgate Plax was added. In group 3, Hiora Himalaya was added. In group 4, Oral B was added. In group 5, Listerine was added. In group 6, Pepsodent was added. Colony-forming units (CFUs) were assessed using a colony counter every six, 24, 48, and 120 hours. After obtaining the pH and CFU of all 72 specimens, software known as the Statistical Package for Social Sciences (SPSS) (IBM Corp., Armonk, NY) was used to analyze the data.

Results

*Candida albicans* adhered to heat-cured denture base acrylic resin sheets differed significantly in response to commercially available mouthwashes (Oral B, Colgate Plax, and Pepsodent) and non-commercial mouthwashes (Hiora Himalaya and Listerine) that contained cetylpyridinium chloride.

Conclusions

Compared to other mouthwashes that do not contain cetylpyridinium chloride (Listerine and Hiora Himalaya), mouthwashes with cetylpyridinium chloride as the active ingredient (Oral B, Pepsodent, and Colgate Plax) have shown good antifungal properties against the adhering *Candida albicans* on denture base resin.

## Introduction

Over the past few decades, prosthodontics has developed into a specialty that has made fixed dental prostheses a reality for everyone. Despite these advancements in implant-supported fixed prostheses, the majority of patients still favor conventional dentures because of their low cost and non-invasive nature [1]. Acrylic resin is used to make these traditional dentures. The incidence of opportunistic fungal infections illnesses has increased, with a particular focus on individuals who wear dentures [2,3]. According to epidemiological research, denture stomatitis affects about 70% of those who wear removable dentures [4,5]. The most significant and common oral fungal pathogen is *Candida albicans*, which has been proven to be associated with this disease [6]. Inflammation and erythema of the oral mucosa that comes into direct contact with the denture are the hallmarks of denture stomatitis [7]. The main etiological factor for this disorder is poor denture hygiene. Additional etiological causes include denture plaque, bacterial and yeast contamination, continuous trauma from poorly fitting dentures, and continuous nighttime wear [8]. Patients with diabetes and those taking steroids orally or through inhalers are more vulnerable. Due to their deteriorating health, elderly patients frequently struggle to maintain denture cleanliness [9]. They, therefore, belong to a high-risk category for denture stomatitis. Since *Candida* colonization is a typical oral cavity condition, it cannot be prevented. Antifungal medication is a very successful treatment for denture stomatitis. However, the problem may return if denture hygiene maintenance is neglected [10].

Chemical materials and mechanical procedures are the two main categories into which commercial denture cleaning products can be divided [11]. Although the mechanical method is thought to be successful at cleaning dentures, it is not preferred by elderly patients [12]. Much research has been conducted on the anti-fungal efficiency of various mouthwashes, but few have evaluated the performance of mouthwashes against *Candida albicans* adherent to acrylic resin [11,13]. The majority of commercially available chemical cleansers are relatively inefficient [14]. Geriatric or differently abled denture users can benefit if mouthwash can be used as an efficient antifungal agent against *Candida albicans* adherent to denture foundation acrylic resins. Therefore, this study aims to evaluate, in vitro, the effectiveness of the three different commercially available mouthwashes in removing *Candida albicans* attached to the heat-cured acrylic denture base and, hence, pave a future way to avoid *Candida* infections. The study aims were designed to know the efficacy of the commercially available mouthwashes and how they can help avoid one of the common oral infections in humans, i.e., *Candida albicans*. Using mouthwash is pretty easy, and it would scientifically pave the way for dental practitioners to prescribe it to their patients, making the study clinically more credible and relevant.

## Materials and methods

Study design

This in vitro study was conducted in the Department of Microbiology at the MES Medical College, Perinthalmanna, Kerala, India. This study was approved by the Institutional Ethical Committee (RES/MDS/2022/MICRO). A total of 72 heat-cured acrylic resin sheets, size 10 × 10 × 2 mm, were fabricated and used for the study.

Data sources and variables

Fabrication of Acrylic Sheet Specimen

A 10 × 10 × 2 mm wax design was created with Hindustan modeling wax. After that, a mold cavity was created, and this wax design was invested and dewaxed in a Varsity flask using plaster of Paris as the investment medium. A sheet of acrylic measuring 10 × 10 × 2 mm was obtained by packing and curing DPI heat-cured acrylic resin into this mold cavity. Seventy-two acrylic sheets were fabricated using the same uniform procedure. There was no polishing of the acrylic sheets to replicate the interior surface of the full denture. After that, all 72 sheets were submerged in distilled water to promote maximum water sorption, which would help to avoid distortion and get rid of any leftover monomer. After that, acrylic sheets were cleaned with a 4% chlorhexidine scrub.

Preparing the Candida albicans Culture

**Figure 1:**
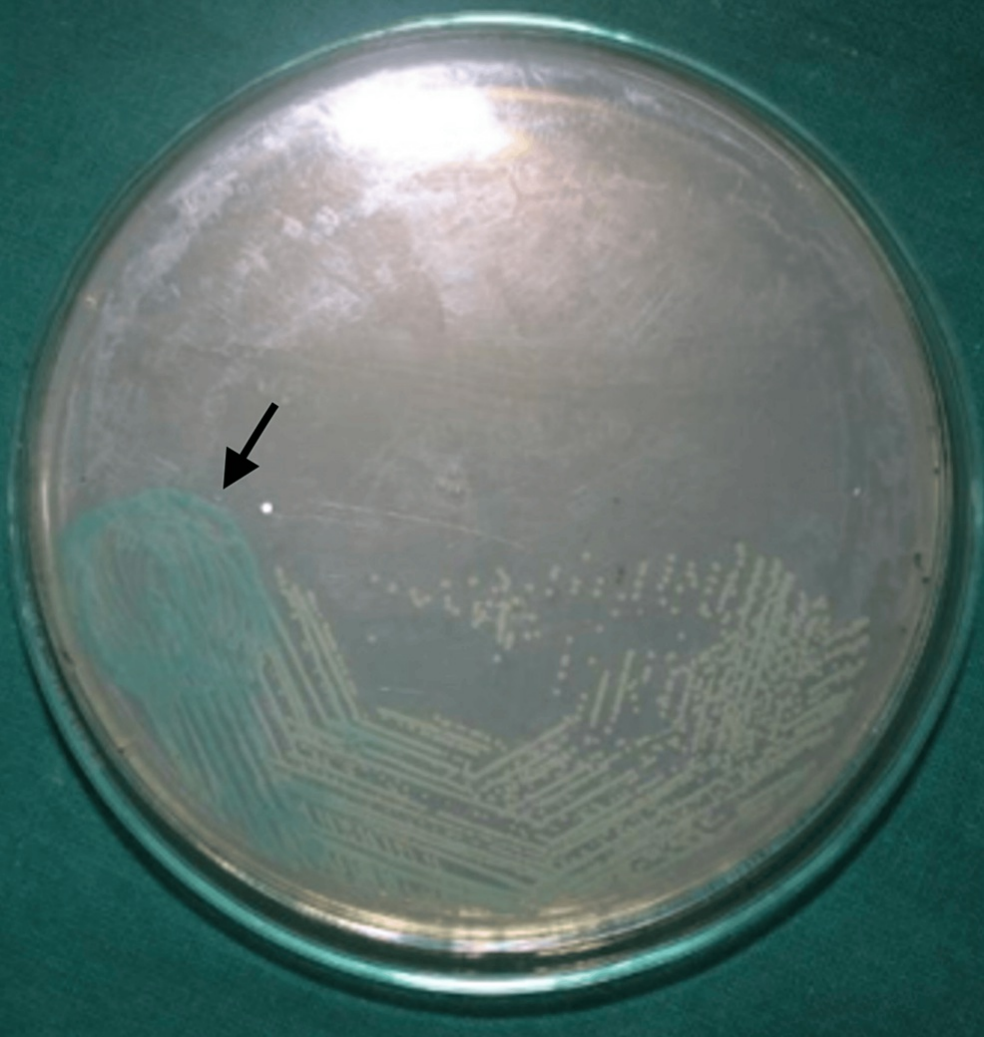
Candida albicans (American Type Culture Collection) on chrome agar

**Figure 2:**
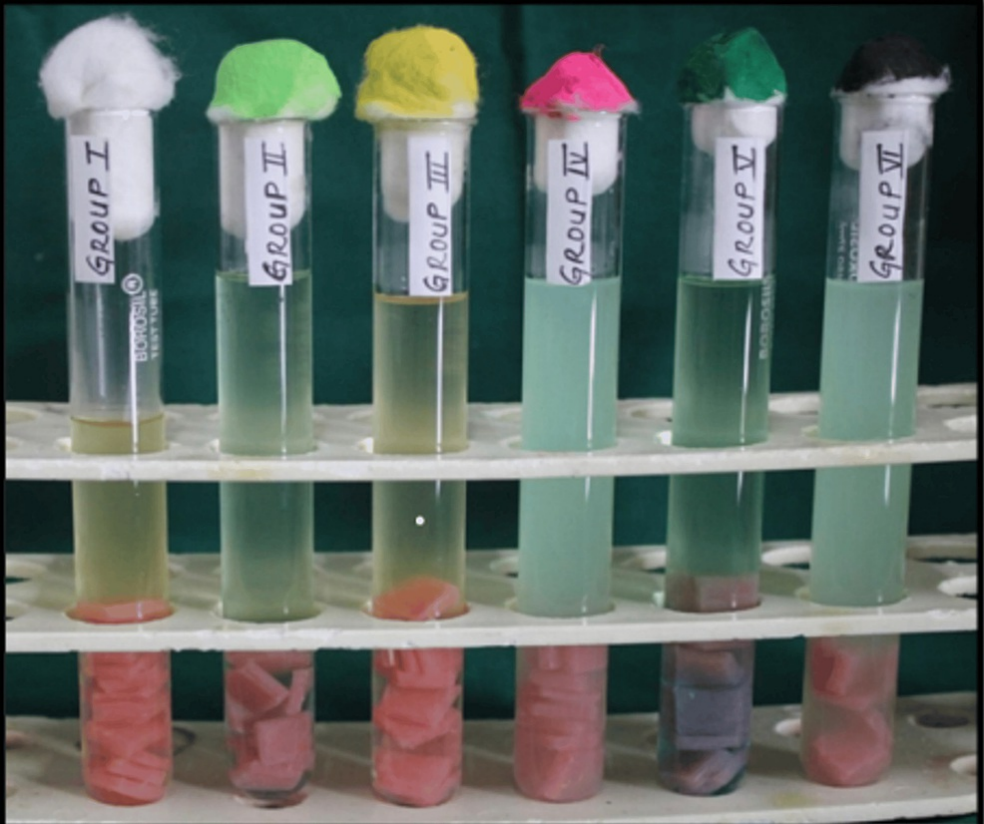
Acrylic sheets divided into six groups and respective mouthwash added in equal quantity Group 1, control group; group 2, Colgate Plax; group 3, Hiora Himalaya herbal mouthwash; group 4, Oral B mouthwash; group 5, Listerine mouthwash; group 6, Pepsodent mouthwash.

The American Type Culture Collection's standard strain of *Candida albicans* was utilized to create the *Candida* strains needed for the investigation (Figure 1). In a flask with a flat bottom, 200 mL of distilled water and 7.42 g of Brain Heart Infusion Broth (BHI Broth) Himedia were combined to create the BHI Broth solution. After that, the BHI Broth solution is autoclaved. Standard strains of *Candida albicans* are thereafter added to the sterilized broth solution via inoculation. A 0.5 McFarland scale was used for the suspension of *Candida albicans* cells. All 72 acrylic sheets were added to this suspension, and they were then incubated for 24 hours at 37º C. Following a 24-hour incubation period, the 72 acrylic sheets were randomized into six groups, consisting of 12 acrylic sheets each: group 1, group 2, group 3, group 4, group 5, and group 6 (Figure 2). Group 1 is the control group; no mouthwash was given to them. Colgate Plax was added to group 2. Hiora Himalaya herbal mouthwash was added to group 3. The Oral B was added to group 4. Listerine was added to group 5. Pepsodent mouthwash was added to group 2. Every mouthwash was added in the same amount. The test tubes were put in the incubator once the mouthwashes had been added to the appropriate groups.

Procedure for the Determination of CFU

**Figure 3:**
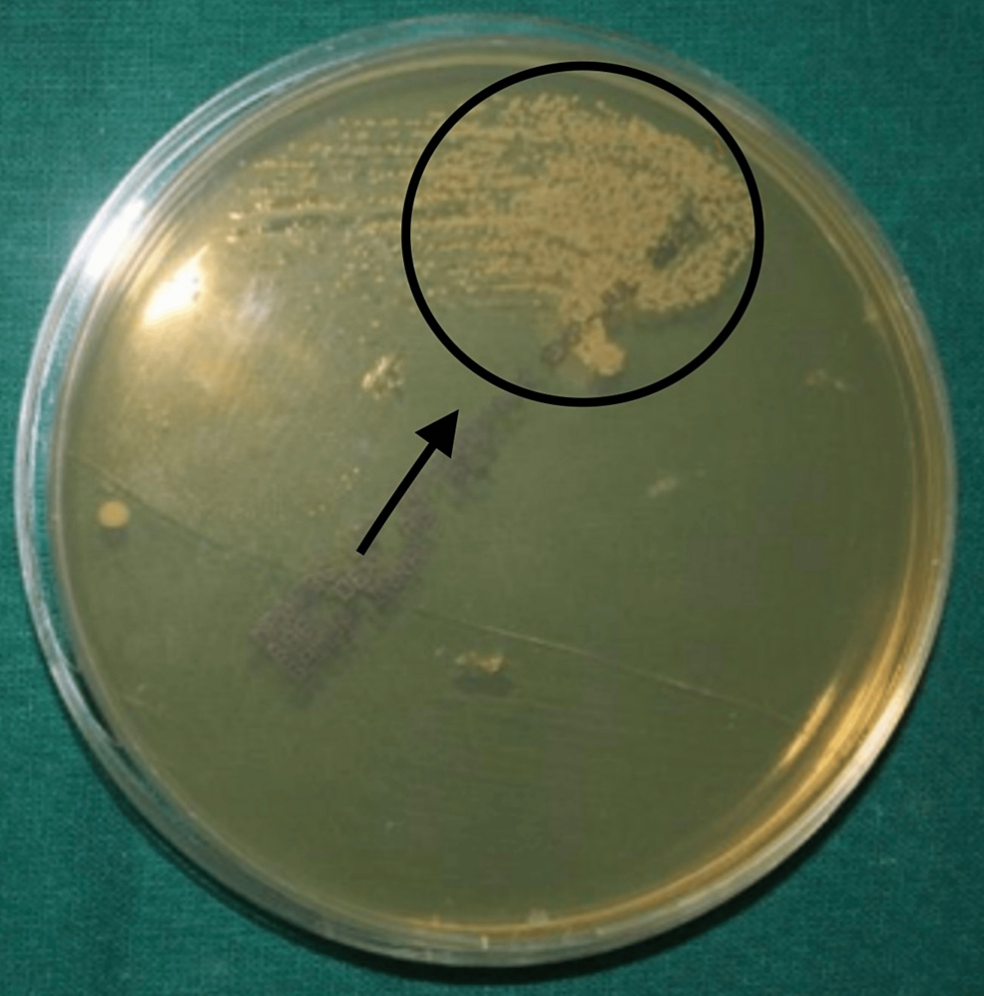
Candida albicans growth on Sabouraud dextrose agar plate

Following a 6-hour incubation period, three acrylic sheets were selected at random from each group and placed in separate test tubes filled with BHI Broth. This resulted in the formation of subgroups, which were designated as follows: group 1, 6 hours A; group 1, 6 hours B; group 1, 6 hours C, etc. Next, the subgroups are incubated independently for a full day at 37ºC. After a 24-hour incubation period, the solution from each test tube was extracted using an inoculating nichrome wire loop with a 4 mm diameter and a 0.01 mL capacity. The solution was then inoculated on the agar plates in a strictly aseptic setting with the aid of a safe laminar airflow cabinet. Streaks were created using the loops in the agar plate (Figure 3). After that, the plates were incubated at 37ºC for 24 hours. The colony-forming units (CFUs) were counted using the colony counter. The colony counts obtained were tabulated.

Procedure for the Determination of pH

Following the addition of mouthwashes, three separate observers measure the pH of each major group medium (Figure 2) at the following intervals using a digital pH meter: every six, 24, 48, and 120 hours. Next, the readings were collected.

Data analysis

All statistical techniques were performed using the Statistical Package for Social Sciences (SPSS) 20.0 (IBM Corp., Armonk, NY). The power (80%) of the study was calculated before the start of the investigation. The mean and standard deviation are used to express all quantitative variables. The quantitative data's assumed normalcy was tested using the Shapiro-Wilk test. The least significant difference (LSD) post hoc test was employed after one-way ANOVA to determine the relationship between the variables. If the probability value was less than 0.05 (p < 0.05), it was deemed statistically significant.

## Results

At 6 hours of incubation, the observations were that all the groups studied showed a decrease in the pH of the medium at 6 hours of incubation with significant differences (p < 0.001) among the medium of various groups. The decrease in pH of the medium was greatest for group 5. Group 1 showed maximum growth of colonies at 6 hours of incubation (147,400 CFU/mL).

There were significant differences between groups 1, 3, and 5 (p < 0.001) in the number of colonies. Groups 2, 4, and 6 did not show any colonies, as shown in Table 1.

**Table 1:** Comparison of colony count on 6 hours of incubation period p < 0.05 is statistically significant; **<0.001 is highly statistically significant; a, b - subgroups.

Groups	Mean ± SD	p Value
Group 1	14,700.00^a,b^ ± 458.25	<0.001**
Group 3	131,933.33^a^ ± 2,437.89	
Group 5	130,833.33^b^ ± 2,764.65	

At 24 hours of incubation, the observations were that all the groups studied showed a decrease in the pH of the medium except groups 2 and 4, which showed a slight increase in pH at 24 hours of incubation. There were significant differences (p < 0.001) in the pH among the medium of various groups. The decrease in pH of the medium was greatest for group 5. Group 1 showed maximum growth of colonies at 24 hours of incubation (148,590 CFU/mL). There were significant differences between groups 1, 3, and 5 (p < 0.001) in the number of colonies. Groups 2, 4, and 6 did not show any colonies, as shown in Table 2.

**Table 2:** Comparison of colony count on 24 hours of incubation period p < 0.05 is statistically significant; **<0.001 is highly statistically significant; a, b - subgroups.

Groups	Mean ± SD	p Value
Group 1	148,590.00^a^ ± 1,046.99	<0.001**
Group 3	122,233.33^a,b^ ± 1,594.78	
Group 5	144,633.33^b^ ± 4,252.45	

At 48 hours of incubation, the observations were that all the groups studied showed a decrease in the pH of the medium at 48 hours of incubation except groups 2 and 6. There were significant differences (p < 0.001) in pH among the medium of various groups. The decrease in pH of the medium was greatest for group 5. Group 1 showed maximum growth of colonies at 48 hours of incubation (153,466 CFU/mL). There were significant differences between groups 1, 3, and 5 (p < 0.001) in the number of colonies. Groups 2, 4, and 6 did not show any colonies. The same has been shown in Table 3.

**Table 3:** Comparison of colony count on 48 hours of incubation period p < 0.05 is statistically significant; **<0.001 is highly statistically significant; a, b - subgroups.

Groups	Mean ± SD	p Value
Group 1	1,543,466.66^a^ ± 3,000.55	<0.001**
Group 3	119,166.66^a,b^ ± 3,197.39	
Group 5	147,733.33^b^ ± 2,638.81	

At 120 hours of incubation, the observations were that all the groups studied showed an increase in the pH of the medium at 120 hours of incubation except group 6, which showed a slight decrease. There were significant differences (p < 0.001) in the pH among the medium of various groups. The lowest pH recorded at 120 hours of incubation was 4.53 (group 5), and the highest pH was 6.7 (group 2). There was a decrease in the number of colonies observed in 120 hours of incubation. Group 1 showed maximum growth of colonies at 120 hours of incubation (149,166.66CFU/mL). There were significant differences between groups 1, 3, and 5 (p < 0.001) in the number of colonies. Groups 2, 4, and 6 did not show any colonies, as shown in Table 4.

**Table 4:** Comparison of colony count on 120 hours of incubation period #p < 0.05 is statistically significant; **<0.001 is highly statistically significant; a, b, and c - subgroups.

Groups	Mean ± SD	p Value
Group 1	149,166.66^a,b^ ± 3,888.87	<0.001**
Group 3	1,111,900.00^a,c ^± 6,109.82	
Group 5	136,800.00^b,c^ ± 5,204.81	

## Discussion

According to a WHO report, the population over 65 is growing at a 2.5% annual pace, compared to a 1.7% annual rate for the entire world's population [14,15]. This indicates that there is a growing need for oral health care for the elderly. Elderly people who wear dentures frequently worry about denture stomatitis [16]. The disorder is closely linked to *Candida* colonization as a result of inadequate denture hygiene management [17,18]. Strict denture hygiene management is the first line of defense against *Candida*-induced denture stomatitis, which presents a challenge for senior patients due to their diminished manual dexterity [19].

For those who have adherent *Candida albicans*, mouthwashes may be a useful solution if they have antifungal properties [20]. The purpose of the current investigation is to determine whether mouthwashes prevent the growth of *Candida albicans*. Additionally, it determines if mouthwash is efficient in removing *Candida albicans* that have attached themselves to denture base resins. It also determines how differently five mouthwashes that are sold commercially perform in terms of inhibiting the growth of *Candida albicans* that are stuck to denture base resins.

Using prefabricated, heat-cured denture base acrylic resin sheets as a substrate, five different commercial mouthwashes were tested for their effects on *Candida albicans* growth. The growth medium's pH and CFUs' growth were monitored at various incubation times. A reduction in the medium's pH signifies the active development of *Candida albicans* and the subsequent generation of acid. The pH of the surrounding medium is lowered by the acid production [9]. According to the results obtained, the null hypothesis was ruled out. The pH change in the medium varied depending on the mouthwash solutions used. *Candida* colonies were obtained from different pH levels of mouthwash solutions. This observation is consistent with the study by Makhira et al. [21] that *Candida albicans* can respond to varying environmental conditions.

The creation of acid by *Candida albicans* during their early growth phase may be the cause of the pH reduction observed in all solutions after 6 hours of incubation. Another possibility for the pH shift is the outcome of combining two different solutions (mouthwash and broth solution). Group 1 had the greatest number of colonies after six hours of incubation. The findings indicate that, after six hours of incubation, the antifungal efficacy of all mouthwashes against adherent *Candida albicans* varied.

At 24 hours of incubation, the decrease in pH and increase in the number of colonies observed in groups 1, 3, and 5 may be due to the exponential growth phase of *Candida albicans*. A comparative increase in pH and no colony formation observed in groups 2 and 4 may be due to the presence of a common active ingredient, cetylpyridinium chloride, which has a known antifungal effect [11,22,23].

After 48 hours of incubation, the persistence of *Candida albicans'* exponential phase of growth can be explained by a drop in pH and an increase in CFU in groups 1, 3, and 5. Groups 2 and 6 maintained their antifungal efficacy by displaying zero CFU/mL and a modest increase in pH. A slight decrease in the pH of group 4 is seen, probably due to the interaction of its various constituents (especially phosphoric acid) with the broth solution [24].

The combined action of active ingredients and the stationary or dying phase of the organism may account for the higher pH and fewer colonies observed in groups 1, 3, and 5 after 120 hours of incubation. By exhibiting no colonies, groups 2, 4, and 6 were able to sustain their potent antifungal action.

The antifungal activity of every mouthwash tested in this study against *Candida albicans* attached to denture base resins varied. This study demonstrates the potent antifungal properties of mouthwashes, including Pepsodent, Colgate Plax, and Oral B. Cetylpyridinium chloride, a common active component in all three of these mouthwashes, has shown antifungal activity [25]. Therefore, mouthwashes containing cetylpyridinium chloride may be an excellent option for people who wear dentures, while further research is needed. Mouthwash was in contact with the examined acrylic resin sheets for six, 24, 48, and 100 hours. Patients do not, however, use mouthwash consistently or for extended periods. This makes this component a study restriction. Further studies can be done with shorter interaction times.

Limitations

Although this study had credible outcomes, it also had a few limitations. A larger sample size can be considered for future studies. Moreover, the eating habits of the patients were not taken into consideration, which could have been influenced by the mouthwash used. This was a single-center study representing a particular demographic and habitat, which could deviate or limit the outcomes. Apart from this, the time duration of usage and the time gap between the two mouthwashes were not taken into consideration. These can be addressed in further studies.

## Conclusions

Mouthwashes, such as Colgate Plax, Oral B, and Pepsodent, showed antifungal activity by increasing pH and reducing the colonies present in the culture medium. Clinicians can provide mouthwash (Colgate Plax, Oral B, and Pepsodent) as a viable option for elderly or differently abled denture wearers who have limited manual dexterity to reduce denture-induced stomatitis. Future studies are required to evaluate the antifungal activity of different types of mouthwash at different concentrations and at small intervals, simulating the oral environment in vitro.
